# Entrapment of high-density grid mapping catheter in a percutaneous ventricular assist device pigtail during ventricular tachycardia ablation with percutaneous hemodynamic support

**DOI:** 10.1016/j.hrcr.2023.04.014

**Published:** 2023-05-02

**Authors:** Robert N. D’Angelo, Serge Korjian, Andre D’Avila, Jonathan W. Waks

**Affiliations:** Harvard-Thorndike Electrophysiology Institute, Division of Cardiovascular Medicine, Beth Israel Deaconess Medical Center, Boston, Massachusetts

**Keywords:** Ventricular tachycardia, Mechanical circulatory support, Electroanatomic mapping, Catheter entanglement, Grid mapping catheter

## Introduction

Patients with incessant ventricular tachycardia (VT), sustained VT despite treatment with antiarrhythmic drugs, or shocks from implantable defibrillators are candidates for catheter ablation (CA).^1^, ^2^, ^3^ Although ablation can be efficacious, mortality is higher in patients with structural heart disease owing to hemodynamic instability from anesthesia and arrhythmia induction.^1^^,^^3^^,^^4^ The PAINESD risk score helps to identify patients at risk of acute hemodynamic decompensation, permitting selection of those likely to benefit from intraprocedural mechanical circulatory support (MCS).^5^ In patients at high risk, temporary MCS, including percutaneous left ventricular (LV) assist devices (pLVADs) and venoarterial extracorporeal membrane oxygenation (VA ECMO) facilitates CA while reducing end-organ hypoperfusion, hemodynamic collapse, and worsening heart failure.^6^^,^^7^ As mapping patients in VT may not be tolerated for extended duration, substrate-based ablation methods have been developed as a complement or alternative to activation and entrainment mapping.^8^ High-density mapping catheters, such as the HD Grid (Abbott, St Paul, MN) and the Optrell (Biosense Webster, Irvine, CA), facilitate high-density mapping and assessment of real-time wavefront propagation. Here, we present a first report of entanglement of an Optrell catheter with an Impella CP (Abiomed, Danvers, MA), suggesting alternative strategies should be taken in patients requiring mechanical support during VT ablation when using grid mapping catheters.

**Key Teaching Points**

• The Optrell catheter (Biosense Webster, Irvine, CA) is a novel grid catheter developed for the mapping of atrial and ventricular arrhythmias, which includes 48 microelectrodes distributed over 6 splines to facilitate high-density mapping and real-time assessment of propagation vectors.
• Use of the Optrell mapping catheter presents a hazard in patients requiring Impella-based mechanical circulatory support (MCS), given risk of entanglement of the mapping catheter with the Impella pigtail.
• In patients requiring Impella-based MCS for ventricular tachycardia ablation, an alternative high-density mapping catheter (Octaray; Biosense Webster, Irvine, CA) or non-pigtail Impella (Impella 5.5; Abiomed, Irvine, CA) should be used.

## Case report

**Figure 1:**
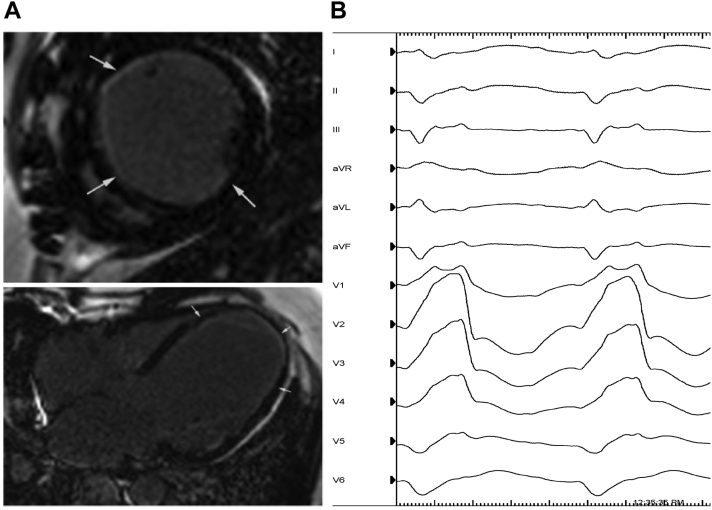
**A:** Induction of clinical ventricular tachycardia (VT) with right bundle, right superior axis, which was localized to the anterior and inferior septum. **B:** Cardiac magnetic resonance imaging demonstrates severe global left ventricular systolic dysfunction with focal partial-thickness subendocardial late gadolinium enhancement in the anterior, anteroseptal, and anterolateral waves, corresponding to region where critical isthmus was mapping in VT, along with fractioned potentials and deceleration zone with right ventricular pacing wavefront.

A 50-year-old male patient with no significant past medical history presented to the hospital with new heart failure with reduced ejection fraction. Transthoracic echocardiogram demonstrated global hypokinesis, LV cavity dilatation, apical aneurysm, and LV ejection fraction of 10%. Coronary angiography demonstrated multivessel coronary artery disease, after which he was deemed not a candidate for coronary artery bypass surgery owing to diffuse coronary disease with poor revascularization targets. Cardiac magnetic resonance imaging showed focal, partial-thickness LV subendocardial late gadolinium enhancement in multiple territories, consistent with prior myocardial infarction (Figure 1A). During the hospitalization, he had frequent premature ventricular contractions with right bundle, left superior axis as well as runs of polymorphic VT. He underwent placement of a single-chamber implantable cardiac defibrillator and was started on goal-directed medical therapy for heart failure.

**Figure 2:**
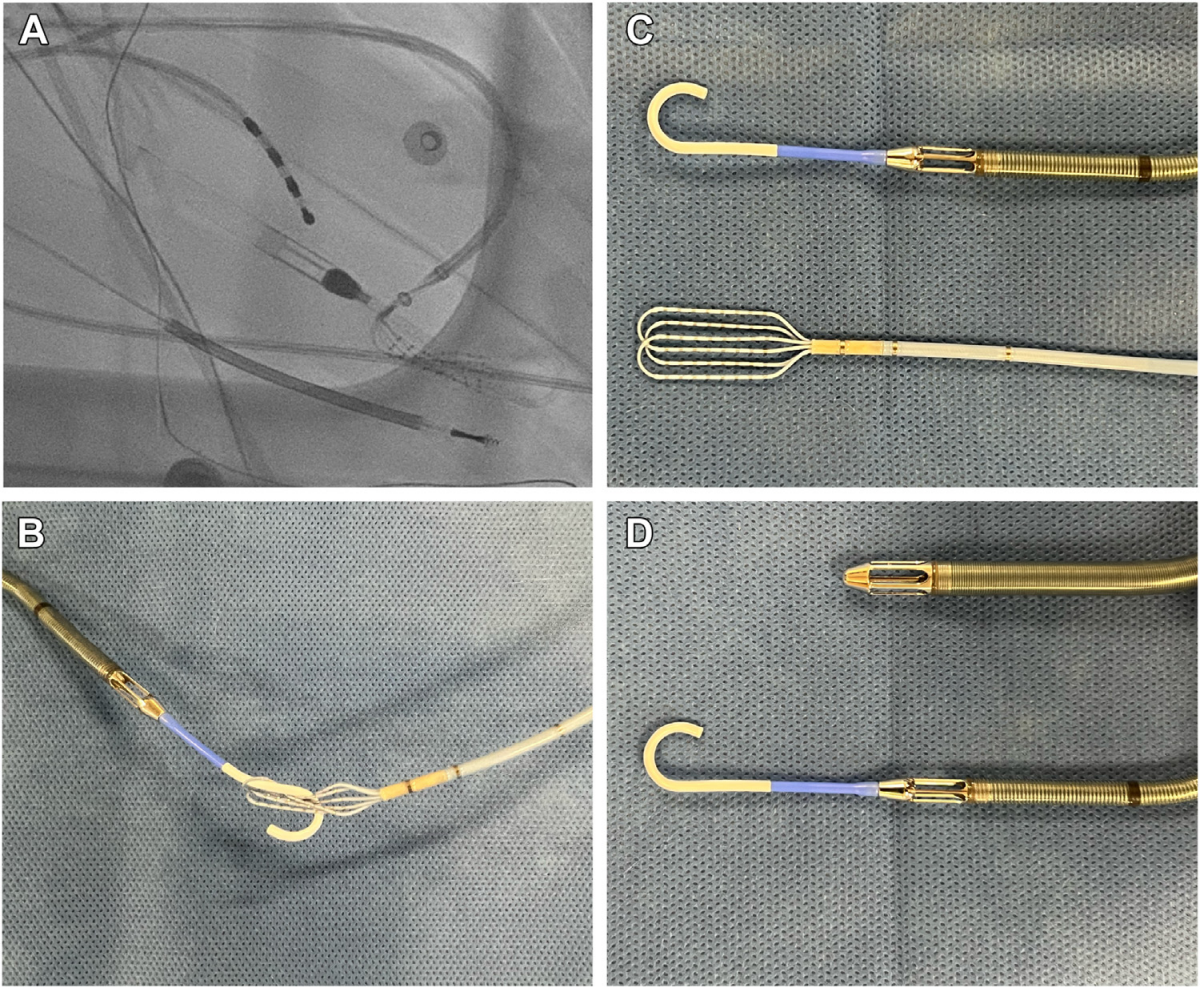
**A:** Fluoroscopy showing entanglement of the Optrell mapping catheter (Biosense Webster, Irvine, CA) and Impella CP (Abiomed, Danvers, MA). The catheter was carefully disentangled under fluoroscopic guidance. **B:** Recreation of catheter entanglement outside the body. **C:** Impella CP (top) along with Optrell mapping catheter (bottom). **D:** The Impella 5.5 (top) can provide up to 5.5 L of mechanical support through axillary cutdown access and does not use a pigtail, compared to the Impella CP (bottom). The Impella CP pigtail poses a risk for entanglement with the Optrell mapping catheter.

After discharge, he had multiple episodes of monomorphic VT, which terminated with antitachycardia pacing. He was started on amiodarone, but developed VT storm treated with multiple shocks from his implantable cardiac defibrillator. He was started on a lidocaine drip and admitted to the cardiac intensive care unit. Given incessant VT, he was intubated, sedated, and underwent stellate ganglion block in preparation for VT ablation. PAINESD score was 18, suggesting high risk for acute hemodynamic decompensation.^5^ Plans were made in consultation with interventional cardiology and the advanced heart failure team for temporary MCS during VT ablation. Based on right-heart catheterization with preserved cardiac output and right ventricular function (right atrium 6 mmHg, right ventricle 30/8 mmHg, pulmonary artery 30/12 mmHg, pulmonary capillary wedge 12 mmHg, cardiac output [Fick] 5.17 L/min, cardiac index 2.7 L/min/m^2^), an Impella CP (Abiomed, Danvers, MA) was selected for MCS during the case. The Impella CP can provide up to 4.3 L/min of support via percutaneous axillary or femoral access (Figure 2D).

On the day of the procedure, the patient was brought to the electrophysiology laboratory. General anesthesia was administrated by a cardiac anesthesiologist with a radial arterial line for continuous blood pressure monitoring. An Impella CP was placed via the left femoral artery. The device was positioned across the aortic valve annulus, with approximately 3.5 cm from the annulus to mid-inlet, and an additional 5.5 cm from mid-inlet to tip of the pigtail catheter.^9^ Transseptal puncture was performed and the LV endocardium was mapped using the Optrell grid catheter and electroanatomic mapping system (CARTO 3, Version 7; Biosense Webster, Irvine, CA).^10^ Given the patient’s tenuous clinical status, a substrate-based ablation approach using isochronal latest activation mapping was planned. While mapping the LV endocardium, the Optrell catheter became difficult to manipulate and was noted to have been entangled in the Impella CP pigtail (Figure 2A and 2B). After several attempts at careful manipulation of the Optrell catheter while holding the Impella fixed, the Optrell catheter was freed from the pigtail and removed from the body without complication (Supplemental Video 1).

The remainder of the LV endocardium was then mapped using an Octaray mapping catheter (Biosense Webster, Irvine, CA) without incident. There was diffusely abnormal bipolar voltage with local abnormal ventricular activity and late potentials along the anterior septum and inferior septum with multiple potential deceleration zones. Given multiple potential conduction channels, the clinical VT (right bundle, right superior axis with cycle length 425 ms) (Figure 1B) was induced, mapped, and successfully ablated. Additional areas were targeted for ablation guided by isochronal latest activation mapping. The patient was noninducible at the end of the procedure.

## Discussion

This is the first reported case of entanglement of Impella and Optrell mapping catheters during VT ablation. Although the mapping catheter was extracted after careful manipulation, this case demonstrates the hazards of using this catheter (and likely other grid-based mapping catheters) with a pigtail-based Impella. A number of studies have shown benefit in supporting patients at high risk for hemodynamic decompensation with MCS during VT ablation.^1^^,^^6^^,^^7^^,^^11^^,^^12^ The Impella CP is frequently used given ease of placement from the femoral artery and up to 4.3 L/min of support. Other options for percutaneous support include the TandemHeart (Cardiac Assist, Inc, Pittsburg, PA), which is inserted through a 21F venous cannula placed transseptally in the left atrium with 17F arterial return cannula as well as VA ECMO. Use of the TandemHeart carries higher risk of vascular complication than the Impella and requires a retrograde approach for mapping and ablation.^13^ Although VA ECMO can provide complete circulatory support, it has a higher risk of vascular complications, it can increase LV afterload, thereby worsening LV function, and is resource intensive.^1^

The Impella CP is placed across the aortic valve annulus as described above and includes a pigtail catheter that sits in the LV.^9^ There is risk of a grid-based mapping catheter becoming entangled with the Impella pigtail, given the spacing of the splines in the paddle design. Notably, there are no reports of similar issues with the similarly designed Advisor HD Grid (Abbott, Abbott Park, IL) mapping catheter, although there are reports of entanglement of this catheter with other diagnostic catheters.^14^ The risk of entanglement with the Impella can be avoided by using the Impella 5.5 (Abiomed, Danvers, MA) (Figure 2D), which does not have a pigtail. The Impella 5.5 can provide an additional support over the Impella CP (up to 5.5 L/min), but requires a surgical axillary artery cutdown or transcaval approach.^9^ Mapping catheters that are not grid/paddle based (such as the Octaray or PentaRay), or a linear ablation catheter, can be used with the Impella CP without risk of entanglement.

In cases where entanglement between a grid catheter and the Impella CP pigtail does occur, holding the Impella stable and then rotating the catheter while adjusting the flex can free the catheters.

## Conclusion

This is the first reported case of Impella CP and Optrell mapping catheter entanglement during VT ablation with MCS. When an Impella CP is used, non-grid-based mapping catheters should be used. If MCS is needed and the electrophysiologist has reasons to use a grid-based mapping catheter such as the Optrell or HD Grid, MCS without a pigtail catheter such as the Impella 5.5, TandemHeart, or ECMO avoids the risk of entanglement, but may increase hospital length of stay and vascular access risk.

## Appendix Supplementary Data

Supplemental Video 1 Disentangling the Optrell grid mapping catheter and the Impella CP pigtail. The first part of the video shows the two catheters entangled. To disentangle the catheters the Optrell catheter was rotated with counter traction on the Impella, followed by advancing the Impella CP and releasing the curve on the Optrell.

## References

[1] Tavazzi G., Dammassa V., Colombo C.N.J. (2022). Mechanical circulatory support in ventricular arrhythmias. Front Cardiovasc Med.

[2] Kushnir A., Pallister K.H., Chaudhary S.B., Cevasco M., Naka Y., Saluja D. (2020). High-density substrate and activation mapping of epicardial ventricular tachycardia during left ventricular assist device implant. HeartRhythm Case Rep.

[3] Mariani S., Napp L.C., Kraaier K. (2021). Prophylactic mechanical circulatory support for protected ventricular tachycardia ablation: a meta-analysis of the literature. Artif Organs.

[4] Cesario D.A., Saxon L.A., Cao M.K., Bowdish M., Cunningham M. (2011). Ventricular tachycardia in the era of ventricular assist devices. J Cardiovasc Electrophysiol.

[5] Muser D., Castro S.A., Liang J.J., Santangeli P. (2018). Identifying risk and management of acute haemodynamic decompensation during catheter ablation of ventricular tachycardia. Arrhythm Electrophysiol Rev.

[6] Miller M.A., Dukkipati S.R., Chinitz J.S. (2013). Percutaneous hemodynamic support with Impella 2.5 during scar-related ventricular tachycardia ablation (PERMIT 1). Circ Arrhythm Electrophysiol.

[7] Aguilar M., Tsao A.L., Croce K.J., Sauer W., Morrow D.A., Tedrow U.B. (2020). Percutaneous right ventricular assist device–supported ventricular tachycardia ablation in a patient with severe right ventricular dysfunction. HeartRhythm Case Rep.

[8] Hawson J., Al-kaisey A., Anderson R.D. (2022). Substrate-based approaches in ventricular tachycardia ablation. Indian Pacing Electrophysiol J.

[9] Zein R., Patel C., Mercado-Alamo A., Schreiber T., Kaki A. (2022). A review of the Impella devices. Interv Cardiol.

[10] Yavin H.D., Bubar Z.P., Higuchi K., Sroubek J., Yarnitsky J., Anter E. (2021). Propagation vectors facilitate differentiation between conduction block, slow conduction, and wavefront collision. Circ Arrhythm Electrophysiol.

[11] Lü F., Eckman P.M., Liao K.K. (2013). Catheter ablation of hemodynamically unstable ventricular tachycardia with mechanical circulatory support. Int J Cardiol.

[12] Bunch T.J., Mahapatra S., Madhu Reddy Y., Lakkireddy D. (2012). The role of percutaneous left ventricular assist devices during ventricular tachycardia ablation. Europace.

[13] Reddy Y.M., Chinitz L., Mansour M. (2014). Percutaneous left ventricular assist devices in ventricular tachycardia ablation multicenter experience. Circ Arrhythm Electrophysiol.

[14] Mar P.L., Chong L., Perez A., Lakkireddy D., Gopinathannair R. (2021). Entrapment of diagnostic catheter within Advisor HD grid mapping catheter. J Cardiovasc Electrophysiol.

